# Correction: A diversity of localized timescales in network activity

**DOI:** 10.7554/eLife.02516

**Published:** 2014-02-18

**Authors:** Rishidev Chaudhuri, Alberto Bernacchia, Xiao-Jing Wang

Chaudhuri R, Bernacchia A, Wang X-J. 2014. A diversity of localized timescales in network activity. *eLife* **3**:e01239. doi: http://dx.doi.org/10.7554/eLife.01239. Published 21 January 2014

The reference number for Xiao-Jing Wang’s Office of Naval Research grant was incorrectly given as ‘N00014-13-1-02’. The correct reference number is ‘N00014-13-1-0297’. The article has been corrected accordingly.

