# Supplementary material for: A diversity of localized timescales in network activity
Source: eLife. 2014 Jan 21;3:e01239. doi: 10.7554/eLife.01239 (PMC3895880; doi:10.7554/eLife.01239)
Supplement: Supplementary file 1. — Mathematical appendix. DOI: http://dx.doi.org/10.7554/eLife.01239.011 [file elife01239s001.pdf]

Mathematical Appendix

1 Translation-independent connectivity implies delocalized eigenvectors

In this section, we show that a linear network with translation-independent connectivity has delocalized eigenvectors, even if the connectivity is strongly localized. This is equivalent to the well-known fact that the eigenvectors of a circulant matrix are the basis functions of a discrete Fourier transform and to Bloch's theorem in solid-state physics [1].

A linear network with N nodes has an N-dimensional coupling matrix, W. If the connectivity profile is translation-independent then $W(j,k) = c(j-k)$, where c is a periodic function with period N. W is thus a circulant matrix.

For an integer n, define $\omega_n = \frac{2\pi n}{N}$. Applying W to the vector $\boldsymbol{v_\lambda} = [e^{i\omega_n}, e^{2i\omega_n}, \ldots, e^{Ni\omega_n}]$ yields

$$\begin{aligned} \left[W\boldsymbol{v_\lambda}\right](j) &= \sum_{k=1}^{N} W(j,k)e^{i\omega_n k} = \sum_{k=1}^{N} c(j-k)e^{i\omega_n k} \\ &= \left(\sum_{p=j-N}^{j-1} c(p)e^{-i\omega_n p}\right) e^{i\omega_n j} \end{aligned}$$

The function in parentheses is periodic with period N, and we shift the limits in the sum to give

$$\left[W\boldsymbol{v_\lambda}\right](j) = \left(\sum_{p=1}^{N} c(p)e^{-i\omega_n p}\right) e^{i\omega_n j} = \lambda e^{i\omega_n j}, \tag{1}$$

where $\lambda = \sum_{p=1}^{N} c(p)e^{-i\omega_n p}$ is the eigenvalue corresponding to $\boldsymbol{v_\lambda}$. So $W\boldsymbol{v_\lambda} = \lambda\boldsymbol{v_\lambda}$ and $\boldsymbol{v_\lambda}$ is an eigenvector of W with eigenvalue $\sum_{p=1}^{N} c(p)e^{-i\omega_n p}$. Note that λ is the Fourier transform of the connectivity profile at frequency ω_n.

$\boldsymbol{v_\lambda}$ has an absolute value of 1 everywhere and so is maximally delocalized. Any network with translation-independent connectivity will show delocalized activity patterns and, because a circulant matrix is normal (i.e. the eigenvalue decomposition is well-conditioned), the results are similar if the connectivity profile is only approximately the same at each node.

2 The functional form of localized eigenvectors from a first-order expansion

We now consider a linear network with a localized connectivity profile that varies between nodes. We explore conditions under which this gives rise to localized eigenvectors and derive the equation for the shape of these eigenvectors (Eq. 2 in the main text).

As in the previous section, we rewrite the connectivity matrix in terms of a relative coordinate $p = j - k$. However, since connectivity now depends on absolute position (and not just the relative separation between nodes), we use an additional variable to index position. Let

$$W(j,k) = c(j, j-k). \tag{2}$$

Thus $c(j,2) = W(j, j-2)$ indexes feedforward projections that span two nodes, and $c(5,p) = W(5, 5-k)$ indexes projections to node 5. For any fixed j, $c(j,p)$ is defined from $p = j - N$ to $p = j - 1$. We extend the definition of $c(j,p)$ to values outside this range by defining $c(j,p)$ to be periodic in p, with the period equal to the size of the network. This is purely a formal convenience to simplify the limits in certain sums and does not constrain the connectivity between the nodes of the network.

Consider the candidate eigenvector $\boldsymbol{v_\lambda}(j) = g_\lambda(j)e^{i\omega j}$. This is a generalization of the eigenvectors in Eq. 1. The dependence of g_λ on j allows the magnitude of the eigenvector to depend on position; setting this function equal to a constant returns us to the translation-independent case. Moreover, note that $g_\lambda(j)$ depends on λ, meaning that eigenvectors corresponding to different eigenvalues (timescales) can have different shapes. For example, different eigenvectors can be localized to different degrees, and localized and delocalized eigenvectors can coexist. ω allows the eigenvector to oscillate across nodes, as in Eq. 1. As before, ω varies between eigenvectors and so depends on λ.

Applying W to $\boldsymbol{v_\lambda}$ yields

$$[W\boldsymbol{v_\lambda}](j) = \sum_{k=1}^{N} W(j,k) g_\lambda(k) e^{i\omega k} = \sum_{k=1}^{N} c(j, j-k) g_\lambda(k) e^{i\omega k} \tag{3}$$

$$= \left(\sum_{p=j-N}^{j-1} c(j,p) g_\lambda(j-p) e^{-i\omega p} \right) e^{i\omega j} \tag{4}$$

Here the term in brackets is no longer independent of j.

So far we have made no use of the requirement of local connectivity and, given that g_λ is an arbitrary function of position and can be different for different timescales, we have placed no constraints on the shape of the eigenvectors. By including an oscillatory term ($e^{i\omega j}$) in our ansatz, we ensure that $g_\lambda(j)$ is constant when connectivity is translation-invariant; this will simplify the analysis.

We now approximate both $c(j,p)$ and $g_\lambda(j-p)$ to first-order (i.e. linearly):

$$\begin{aligned} c(j,p) &\approx c(j_0,p) + f_1(j_0,p)(j-j_0) \\ g_\lambda(j-p) &\approx g_\lambda(j) - f_2(j)p. \end{aligned} \tag{5}$$

Here j_0 is a putative center of the eigenvector. f_1 and f_2 are functions that provide the slope in a linear approximation of c and g_λ. There are many such possibilities and, at the moment, we have placed no constraints on them. In the continuous limit, setting f_1 and f_2 to be the appropriate derivatives yields the best local linear approximation (and the expansion is then a first-order Taylor series).

Substituting Eq. 5 into Eq. 4 we get

$$[W\boldsymbol{v}_\lambda](j) = \left(\sum_{p=j-N}^{j-1} \Big[c(j_0,p) + f_1(j_0,p)(j-j_0) \Big] \Big[g_\lambda(j) - f_2(j)p \Big] e^{-i\omega p} \right) e^{i\omega j} \tag{6}$$

We expect these approximations to be valid only locally. However, if connectivity is local then the major contribution to the sum comes from small values of p. For large values of p, $g_\lambda(j-p)$ is multiplied by connectivity strengths close to 0 and so we only need to approximate g_λ for p close to 0. Similarly, in approximating $c(j,p)$ around $j = j_0$, we expect our approximation to be good in the vicinity of $j = j_0$. However, if our eigenvector is indeed localized around j_0, then $g_\lambda(k)$ is small when $|k - j_0|$ is large. For small p, large values of $|k - j_0|$ approximately correspond to large values of $|j - j_0|$, and so $c(j,p)$ makes a contribution to the sum only when $j \approx j_0$.

The zeroth order term in Eq. 6 is

$$\left(\sum_{p=j-N}^{j-1} c(j_0,p) e^{-i\omega p} \right) g_\lambda(j) e^{i\omega j} = \lambda(j_0,\omega) \boldsymbol{v}_\lambda(j)$$

As before, the function in parentheses is periodic in p with period N (recall that $c(j,p)$ was extended to be periodic in p). Thus to zeroth-order $\boldsymbol{v}_\lambda$ is an eigenvector with eigenvalue

$$\lambda(j_0,\omega) = \sum_{p=1}^{N} c(j_0,p) e^{-i\omega p}. \tag{7}$$

Before proceeding further, we contrast this expression with Eq. 1, which is similar except that $c(j_0,p)$ does not depend on j_0 (translation-invariance). Eq. 1 tells us that the eigenvectors are described by a common functional form ($e^{i\omega j}$), with a parameter (ω) that varies from eigenvector to eigenvector. It also specifies an analytic relation between ω for a particular eigenvector and the corresponding eigenvalue. We seek a similar relationship for the more general case where connectivity depends on position.

For λ to be an exact eigenvalue in Eq. 6, the higher-order terms should vanish. By setting the first-order term in this equation to 0, we obtain an equation for $g_\lambda(j)$:

$$(j - j_0)g_\lambda(j)\sum_p f_1(j_0,p)e^{-i\omega p} = f_2(j)\sum_p pc(j_0,p)e^{-i\omega p} \tag{8}$$

To get a closed-form expression for g_λ, we need explicit forms for f_1 and f_2. If the number of nodes in the network is large, or if we assume that both c and g are sampled from some underlying smooth function (as is the case in our examples), then f_1 and f_2 are naturally chosen to be the derivatives, which are the best local linear approximations (by definition). In this case we set

$$f_1(j_0,p) = \left.\frac{\partial c}{\partial j}\right|_{j_0,p} \quad \text{and} \quad f_2(j) = g'_\lambda(j).$$

Substituting into Eq. 8 gives us a differential equation in g_λ:

$$-\alpha(j_0,\omega)^2 g'_\lambda(j) = (j - j_0)g_\lambda(j) \tag{9}$$

Where

$$\alpha(j_0,\omega)^2 = -\frac{\sum_p pc(j_0,p)e^{-i\omega p}}{\sum_p \left.\frac{\partial c}{\partial j}\right|_{j_0,p} e^{-i\omega p}}. \tag{10}$$

Thus α^2 is a ratio of discrete Fourier transforms at the frequency ω. Note that the denominator is a weighted measure of network heterogeneity at the location j_0. Also note that α^2 can be written in terms of λ as (compare the twist condition of [3]):

$$\alpha^2(j_0,\omega) = -i\frac{\frac{\partial\lambda}{\partial\omega}}{\frac{\partial\lambda}{\partial j_0}}. \tag{11}$$

Solving for g_λ in Eq. 9 yields

$$g_\lambda(j) = C_1 e^{-\frac{(j-j_0)^2}{2\alpha(j_0,\omega)^2}},$$

where C_1 is a constant. Thus, to first-order, the eigenvector is given by the modulated Gaussian function

$$\boldsymbol{v}_\lambda(j) = e^{-\frac{(j-j_0)^2}{2\alpha(j_0,\omega)^2} + i\omega j}. \tag{12}$$

In general, α can be complex. In order for $\boldsymbol{v}_\lambda$ to be localized, $\mathfrak{Re}(\alpha^2)$ must be positive for the corresponding values of j_0 and ω, and we only accept an eigenvector as a valid solution if this is the case. Thus the approach is self-consistent: we assumed that there existed a localized eigenvector, combined this with the requirement of local connectivity to solve for its putative shape, and then restricted ourselves to solutions that did indeed conform to our initial assumption.

In Eq. 6 we expand both g_λ and c to first-order, using functions f_1 and f_2. These functions are somewhat arbitrary, in that they are only required to be good local linear approximations. In the large-N limit (or if $c(j,p)$ is sampled from some underlying continuous function) these are most naturally taken to be the derivatives, since the derivative is the best local linear approximation. Taking f_2 to be the derivative of g_λ has the advantage of giving a differential equation for g_λ, which can be solved to yield a closed-form expression. More generally, though, for f_2 we could use a discrete difference to approximate the changes in g (e.g. $\Delta g = g(j+1) - g(j)$) and attempt to solve the resulting difference equation. On the other hand, the replacement of f_1 by $\frac{\partial c}{\partial j}(j_0, p)$ only serves to help us find closed-form expressions for α^2 and has no consequences for the functional form of Eq. 9.

Finally, we include an oscillatory term (i.e. $e^{i\omega j}$) in our ansatz so that a zeroth-order expansion in connectivity (i.e. connectivity is locally translationally invariant) yields eigenvectors given by a zeroth-order expansion in g (i.e. g_λ is locally constant). This ensures that the zeroth order expansion locally restates the conclusions of Section 1.

Before we proceed to applications of this analysis, it is useful to step back and to clarify what our theory has given us.

Contrast our result with the discussion of Eq. 1 that followed Eq. 7. For a particular class of networks/matrices we have identified a general functional form for eigenvectors (that of a modulated Gaussian), with two parameters (j_0 and ω) that vary from eigenvector to eigenvector. We have also specified an analytic relation between j_0 and ω for a particular eigenvector and the corresponding eigenvalue (or timescale): given an eigenvalue λ, we use Eq. 7 to find the values of j_0 and ω that correspond to λ (note that we're matching two real parameters to one complex value). We use these to calculate α^2. However, because of our initial assumption that the eigenvector under consideration was localized, our result only applies to eigenvectors for which $\alpha^2 > 0$.

For a circulant matrix, the parameter ω_n in Eq. 1 is known and the eigenvalues can be computed from the ω_n. In general, for the matrices we consider, our method doesn't provide a way to analytically compute the eigenvalues and, given that we consider a large class of matrices, such a formula would be unlikely. Instead our theory identifies how the shape of a particular localized eigenvector depends on network architecture and on its corresponding timescale.

While the theory doesn't tell us which timescales a finite network displays (stronger asymptotic results may be possible), it identifies a region of the complex plane within which the eigenvalues of the network lie. This is the range of $\lambda(j_0, \omega)$, swept out by varying j_0 and ω. For each value of j_0 and ω we can compute α^2, and eigenvalues / timescales that lie in the subset of this region where $\alpha^2 > 0$ correspond to localized eigenvectors (this is equivalent to the twist condition in [3]). Thus our theory tells us where the onset of localization lies and how changing the network parameters changes the boundary between regions of localized and delocalized timescales. If α^2 is always positive then all eigenvalues are localized. Moreover, the values of j_0 at which α^2 is positive tell

us the nodes that can potentially host localized timescales, which can be useful in analyzing networks with a mixture of localized and delocalized timescales. For an example of such a network, see Figure 3 - Figure Supplement 1.

The theory tells us that, to first order, localized eigenvectors are modulated Gaussians with two parameters that depend both on the connectivity architecture and on the particular eigenvalue/timescale under consideration. Some eigenvectors/timescales are localized (i.e. have a positive width parameter/α^2) and others are delocalized (have a negative α^2). Both the existence of localization and the width of the localized eigenvectors can depend on timescale and thus to predict the shape of a particular eigenvector we need to know the timescale it corresponds to. However, once we know this timescale we have an analytic expression for how the eigenvector shape depends on network parameters and how changing network parameters promotes or hinders localization (of course, changing network parameters will also change the eigenvalue but if these are changed continuously then Eq. 7 should tell us how the eigenvalue changes locally).

In a number of cases, we can draw further conclusions about the shapes of all localized eigenvectors in a network without computing the eigenvalues. For example, in the network of Fig. 3 (analyzed further in Section 3 below), the eigenvector width does not depend on the location of the eigenvector. This is a special example but not an unnatural one; a gradient of local properties is one of the simplest deterministic ways to break translation-invariance, and as such is particularly interesting theoretically. For the network of Fig. 4 (see Section 4 below), the eigenvectors are all localized and the width depends only weakly on position. Such simplifications are possible because eigenvector width depends on j_0 and ω (Eq. 10) in a relatively simple way.

If there are constraints on the spectrum / timescales of the network, then the theory allows us to transfer this into constraints on the shapes of the corresponding eigenvectors. For example, if a network has low-rank connectivity then the spectrum of the corresponding matrix has a cluster of eigenvalues around the intrinsic timescale of each node. As another example, models with connectivity that decays very rapidly with distance will tend to have eigenvalues clustered around the real axis (with distance bounded by Gerschgorin disks), which will force ω to be close to 0 or π. This is also approximately true for the network architecture of Fig. 3, where the eigenvalues lie on the real line (in the case with only nearest-neighbor connections this is exact and can be shown with a similarity transform).

Finally, the analysis reveals the qualitative factors that are important for a network to localize (i.e. breaking translation-invariance, asymmetry), and can help in conceiving of possible network architectures. The first example (Fig. 3) emerged from an attempt to understand the origins of timescale localization in a model of large-scale interactions in the cortex. The second network (Fig. 4) was designed from a calculation requiring the width of localized eigenvectors to be independent of ω.

3 Localized eigenvectors for a network with a gradient of self-coupling strengths

We now apply the theory from the previous section to derive Eq. 4 in the main text.

Recall that the connectivity for this architecture is given by

$$W(j,k) = \begin{cases} \mu_0 + \Delta_r j & \text{for } j = k \\ \mu_f e^{-(j-k)/l_c} & \text{for } j > k \\ \mu_b e^{(j-k)/l_c} & \text{for } j < k \end{cases} \tag{13}$$

Define $p = j-k$ and rewrite the connectivity in terms of a relative coordinate $c(j,p) = W(j,j-p)$

$$c(j,p) = \begin{cases} \mu_0 + \Delta_r j & \text{for } p = 0 \\ \mu_f e^{-p/l_c} & \text{for } p > 0 \\ \mu_b e^{p/l_c} & \text{for } p < 0 \end{cases} \tag{14}$$

From Eq. 7 the eigenvalues are

$$\lambda(j_0,\omega) = \mu_0 + \Delta_r j_0 + \mu_f \sum_{p=1}^{j_0-1} e^{-p/l_c} e^{-pi\omega} + \mu_b \sum_{p=-1}^{j_0-N} e^{p/l_c} e^{-pi\omega}, \tag{15}$$

For a particular eigenvalue λ, we solve this equation to give j_0 and ω for the corresponding eigenvector. We then use Eq. 10 to calculate α^2.

$$\alpha^2 = -\frac{\sum_p pc(j_0,p)e^{-i\omega p}}{\sum_p \frac{\partial c}{\partial j}\Big|_{j_0,p} e^{-i\omega p}} = -\frac{\mu_f \sum_{p=1}^{j_0-1} pe^{-p/l_c}e^{-pi\omega} + \mu_b \sum_{p=-1}^{j_0-N} pe^{p/l_c}e^{-pi\omega}}{\Delta_r}. \tag{16}$$

The sums in Eq. 15 are terms in a geometric series, and can be solved in closed form:

$$\sum_{p=1}^{j_0-1} e^{-p/l_c} e^{-pi\omega} = \frac{e^{-1/l_c - i\omega} - e^{-j_0(1/l_c + i\omega)}}{1 - e^{-1/l_c - i\omega}}$$

$$\sum_{p=-1}^{j_0-N} e^{p/l_c} e^{-pi\omega} = \frac{1 - e^{-(N-j_0)(1/l_c - i\omega)}}{e^{1/l_c - i\omega} - 1}$$

Furthermore, for most values of j_0, $e^{-j_0(1/l_c+i\omega)}$ and $e^{-(N-j_0)(1/l_c-i\omega)}$ are small (especially if connectivity is localized) and can be ignored, so that

$$\sum_{p=1}^{j_0-1} e^{-p/l_c} e^{-pi\omega} \approx \frac{e^{-i\omega}}{e^{1/l_c} - e^{-i\omega}}$$

$$\sum_{p=-1}^{j_0-N} e^{p/l_c} e^{-pi\omega} \approx \frac{e^{i\omega}}{e^{1/l_c} - e^{i\omega}}$$

Substituting allows us to simplify Eq. 15 yielding

$$\lambda(j_0, \omega) \approx \mu_0 + \Delta_r j_0 + \mu_f \frac{e^{-i\omega}}{e^{1/l_c} - e^{-i\omega}} + \mu_b \frac{e^{i\omega}}{e^{1/l_c} - e^{i\omega}},$$

and noting that the sums in Eq. 16 are derivatives of those in Eq. 15 gives

$$\alpha^2 = \frac{(\mu_b - \mu_f)\left(-1 + \cos(\omega)\cosh\left(\frac{1}{l_c}\right)\right) + i(\mu_b + \mu_f)\sin(\omega)\sinh\left(\frac{1}{l_c}\right)}{2\Delta_r\left(\cos(\omega) - \cosh\left(\frac{1}{l_c}\right)\right)^2}.$$

By matching the eigenvalues to j_0 and ω we find that $\omega = \pi$, so that the equation for α^2 can be further simplified to give:

$$\alpha^2 = \frac{\mu_f - \mu_b}{2\Delta_r\left(1 + \cosh(\frac{1}{l_c})\right)} \tag{17}$$

4 Localized eigenvectors for a network with a gradient of connectivity decay lengths

Recall that the connectivity is

$$W(j,k) = \begin{cases} \mu_0 & \text{for } j = k \\ \mu_f e^{-(f_0 + f_1 k)(j-k)} & \text{for } j > k \\ \mu_b e^{(b_0 - b_1 k)(j-k)} & \text{for } j < k \end{cases} \tag{18}$$

Defining $p = j - k$ and rewriting in terms of $c(j,p) = W(j, j-p)$ we get

$$c(j,p) = \begin{cases} \mu_0 & \text{for } p = 0 \\ \mu_f e^{-(f_0 + f_1 (j-p))p} & \text{for } p > 0 \\ \mu_b e^{(b_0 - b_1 (j-p))p} & \text{for } p < 0 \end{cases} \tag{19}$$

Using Eq. 7 the eigenvalues are

$$\lambda(j_0, \omega) = \mu_0 + \mu_f \sum_{p=1}^{j_0-1} e^{-(f_0+f_1(j_0-p))p} e^{-pi\omega} + \mu_b \sum_{p=-1}^{j_0-N} e^{(b_0-b_1(j_0-p))p} e^{-pi\omega} \tag{20}$$

We can calculate α^2 as in Eqs. 10 or 11. While we will typically do this numerically, we can get an approximate closed-form expression by noting that, as a consequence of local connectivity, most of the contribution to the sums in Eq. 20 comes from small values of p and, provided that j_0 is not too small, we can replace $j_0 - p$ by $j_0 \pm 1$ to get

$$\sum_{p=1}^{j_0-1} e^{-(f_0+f_1(j_0-p))p} e^{-pi\omega} \approx \sum_{p=1}^{j_0-1} e^{-(f_0+f_1(j_0-1))p} e^{-pi\omega}$$

$$\sum_{p=-1}^{j_0-N} e^{(b_0-b_1(j_0-p))p} e^{-pi\omega} \approx \sum_{p=-1}^{j_0-N} e^{(b_0-b_1(j_0+1))p} e^{-pi\omega}.$$

These are geometric series and can be solved in closed-form. As an added simplification, note that as in the previous section,

$$\sum_{p=1}^{j_0-1} e^{-(f_0+f_1(j_0-1))p/l_c} e^{-pi\omega} \approx \sum_{p=1}^{\infty} e^{-(f_0+f_1(j_0-1))p/l_c} e^{-pi\omega} \approx \frac{1}{-1+e^{f_0+f_1(j_0-1)+i\omega}}$$

$$\sum_{p=-1}^{j_0-N} e^{(b_0-b_1(j_0+1))p/l_c} e^{-pi\omega} \approx \sum_{p=-1}^{-\infty} e^{(b_0-b_1(j_0+1))p/l_c} e^{-pi\omega} \approx \frac{1}{-1+e^{b_0-b_1(j_0+1)-i\omega}}.$$

With this approximation we get

$$\alpha^2 = \frac{\mu_f \gamma_2(j_0,\omega) - \mu_b \gamma_1(j_0,\omega)}{\mu_f f_1 \gamma_2(j_0,\omega) - \mu_b b_1 \gamma_1(j_0,\omega)} \tag{21}$$

where

$$\gamma_1(j,\omega) = \cosh(f_0 + f_1(j-1) + i\omega) - 1$$
$$\gamma_2(j,\omega) = \cosh(b_0 - b_1(j+1) - i\omega) - 1$$

The theoretical predictions plotted in Fig. 4 are from this approximation.

5 The functional form of localized eigenvectors from a second-order expansion

In Section 2 we expanded both $c(j,p)$ and $g_\lambda(j)$ to first-order, and solved for the functional form of g_λ. However, as shown in Fig. 3, this approximation breaks down for the architecture of Eq. 13 when $\mu_f - \mu_b$ becomes small. Here we improve the approximation by extending it to second-order in g_λ.

As before, consider a candidate eigenvector $\boldsymbol{v_\lambda}(j) = g_\lambda(j)e^{i\omega j}$ and apply the matrix W to get

$$[W\boldsymbol{v_\lambda}](j) = \sum_{k=1}^{N} W(j,k) g_\lambda(k) e^{i\omega k} = \sum_{k=1}^{N} c(j, j-k) g_\lambda(k) e^{i\omega k}$$
$$= \left(\sum_{p=j-N}^{j-1} c(j,p) g_\lambda(j-p) e^{-i\omega p} \right) e^{i\omega j}$$

We again approximate both $c(j,p)$ and $g_\lambda(j-p)$, but carry the approximation of g_λ to second-order:

$$c(j,p) = c(j_0,p) + \left.\frac{\partial c}{\partial j}\right|_{j_0,p} (j - j_0)$$
$$g_\lambda(j-p) = g_\lambda(j) - p g'_\lambda(j) + \frac{p^2}{2} g''_\lambda(j)$$

For simplicity, we've written the coefficients in the expansions of c and g_λ in terms of their derivatives (i.e. as Taylor series); this is exact in the continuous limit (see the discussion of this issue in section 2).

Substituting these approximations gives

$$[W\boldsymbol{v_\lambda}](j) = \left(\sum_{p=j-N}^{j-1} \left[c(j_0,p) + \left.\frac{\partial c}{\partial j}\right|_{j_0,p} (j-j_0) \right] \left[g_\lambda(j) - p g'_\lambda(j) + \frac{p^2}{2} g''_\lambda(j) \right] e^{-i\omega p} \right) e^{i\omega j}$$

The zeroth order term gives us the eigenvalue:

$$\lambda(j_0,\omega) = \sum_{p=1}^{N} c(j_0,p) e^{-i\omega p}.$$

To make this exact we require the higher-order terms to vanish. When the second-order term is small, then this is equivalent to requiring that the first-order term vanish and this gave us a first-order differential equation for g_λ (Eq. 9). If, however, the first-order expansion does a poor job of capturing the behavior of the eigenvector around the point of interest, then we require the sum of the first and second-order terms to vanish, giving a second-order differential equation in g_λ.

$$\left(\sum_p \frac{p^2}{2} c(j_0,p) e^{-i\omega p} \right) g''_\lambda(j) - \left(\sum_p p \left[c(j_0,p) + \left.\frac{\partial c}{\partial j}\right|_{j_0,p} (j-j_0) \right] e^{-i\omega p} \right) g'_\lambda(j) + \left(\sum_p \left.\frac{\partial c}{\partial j}\right|_{j_0,p} e^{-i\omega p} \right) (j-j_0) g_\lambda(j) = 0 \tag{22}$$

The coefficients in Eq. 22 are linear in j. If we had expanded $c(j,p)$ to second-order, these coefficients would be quadratic in j. The coefficients also involve sums over p. Since these do not depend on j, they will not affect the form of the differential equation. However, they encode the dependence of the parameters of the solution on the parameters of the connectivity and so it will be useful to calculate them in closed form. In the next section, we solve Eq. 22 for a special case.

6 Second-order expansion for a network with a gradient of self-coupling strengths

The solutions to Eq. 22 are typically not Gaussian and take various functional forms. Here we solve for the family of solutions that arise when connectivity is translationally invariant except for self-couplings (i.e. the matrix varies along the main diagonal).

The constraint that connectivity is translationally invariant except along the main diagonal is equivalent to requiring that $\frac{\partial c(j,p)}{\partial j} = 0$ when $p \neq 0$. This simplifies Eq. 22 to yield

$$\left(\sum_p \frac{p^2}{2} c(j_0,p) e^{-i\omega p}\right) g''_\lambda(j) - \left(\sum_p p c(j_0,p) e^{-i\omega p}\right) g'_\lambda(j) + \left.\frac{\partial c}{\partial j}\right|_{j_0,0} (j - j_0) g_\lambda(j) = 0 \tag{23}$$

For notational convenience, abbreviate the parameters in this equation as

$$\sum_p \frac{p^2}{2} c(j_0,p) e^{-i\omega p} = a_1, \quad -\sum_p p c(j_0,p) e^{-i\omega p} = a_2 \quad \text{and} \quad \left.\frac{\partial c}{\partial j}\right|_{j_0,0} = a_3.$$

Define

$$\beta_1 = -\frac{a_3}{a_1} \quad \text{and} \quad \beta_2 = -\frac{a_2}{2a_1}$$

The solution to Eq. 23 is then

$$C_1 e^{\beta_2(j-j_0)} \mathrm{Ai}\left(\frac{\beta_1(j-j_0) + \beta_2^2}{\beta_1^{2/3}}\right) + C_2 e^{\beta_2(j-j_0)} \mathrm{Bi}\left(\frac{\beta_1(j-j_0) + \beta_2^2}{\beta_1^{2/3}}\right) + \tag{24}$$

Here Ai and Bi are Airy functions[2] and C_1 and C_2 are constants. Bi(x) diverges supra-exponentially as $x \to \infty$, and falls off slowly as $x \to -\infty$ [2] and so a localized eigenvector will only involve the Ai(x) term. Thus we have

$$g_\lambda(j) = C_1 e^{\beta_2(j-j_0)} \mathrm{Ai}\left(\frac{\beta_1(j-j_0) + \beta_2^2}{\beta_1^{2/3}}\right) \tag{25}$$

Note that this function is asympotically less localized than the Gaussians from a first-order expansion, which decay as e^{-j^2}: for increasing j, the function falls off as $e^{-kj^{3/2}}$, while for decreasing j it falls off exponentially. Thus far we have not assumed any particular functional form for connectivity, since the functional form only changes the parameters in Eq. 25. We now use the connectivity of Eq. 13 to derive Eq. 5 in the main text. Recall that

$$c(j,p) = \begin{cases} \mu_0 + \Delta_r j & \text{for } p = 0 \\ \mu_f e^{-p/l_c} & \text{for } p > 0 \\ \mu_b e^{p/l_c} & \text{for } p < 0 \end{cases}$$

And the eigenvalue is

$$\lambda(j_0,\omega) = \mu_0 + \Delta_r j_0 + \mu_f \frac{e^{-i\omega}}{e^{1/l_c} - e^{-i\omega}} + \mu_b \frac{e^{i\omega}}{e^{1/l_c} - e^{i\omega}}.$$

Let

$$\begin{aligned} \sigma &= \mu_f \sum_{p=1}^{\infty} e^{-p/l_c} e^{-pi\omega} + \mu_b \sum_{p=1}^{\infty} e^{-p/l_c} e^{pi\omega} \\ &= \mu_f \frac{e^{-i\omega}}{e^{1/l_c} - e^{-i\omega}} + \mu_b \frac{e^{i\omega}}{e^{1/l_c} - e^{i\omega}} \end{aligned}$$

Then $a_2 = -i\frac{\partial \sigma}{\partial \omega}$ and $a_1 = -\frac{1}{2}\frac{\partial^2 \sigma}{\partial \omega^2}$.

Calculating these derivatives and setting $\omega = \pi$ (see discussion in section 3) we find that

$$a_1 = \frac{e^{\frac{1}{l_c}}\left(1 - e^{\frac{1}{l_c}}\right)(\mu_b + \mu_f)}{2\left(1 + e^{\frac{1}{l_c}}\right)^3}, \quad a_2 = \frac{e^{\frac{1}{l_c}}(\mu_f - \mu_b)}{\left(1 + e^{\frac{1}{l_c}}\right)^2} \quad \text{and} \quad a_3 = \Delta_r.$$

Consequently

$$\beta_1 = \frac{\Delta_r \text{Csch}\left(\frac{1}{2l_c}\right)^4 \text{Sinh}\left(\frac{1}{l_c}\right)^3}{(\mu_b + \mu_f)} \quad \text{and} \quad \beta_2 = \frac{(\mu_f - \mu_b)\text{Coth}\left(\frac{1}{2l_c}\right)}{(\mu_f + \mu_b)}$$

As $\mu_f - \mu_b \to 0$, β_2 becomes small and the exponential in Eq. 25 becomes increasingly flat. This delocalizes the leading edge of the eigenvector (see Fig. 3).

References

[1] N. W. Ashcroft and D. N. Mermin. *Solid State Physics*. Rinehart and Winston, New York, 1976.

[2] F. W. J. Olver. Chapter 9 Airy and Related Functions. In F. W. J. Olver, D. W. Lozier, R. F. Boisvert, and C.W. Clark, editors, *NIST Handbook of Mathematical Functions*, pages 193–214. Cambridge University Press, New York, 2010.

[3] L. N. Trefethen and M. Embree. *Spectra and Pseudospectra: The Behavior of Nonnormal Matrices and Operators*. Princeton University Press, Princeton, 2005.
